# Challenge in diagnosis of late onset necrotizing enterocolitis in a term infant: a case report

**DOI:** 10.1186/s12887-021-02626-y

**Published:** 2021-03-30

**Authors:** Gunadi, Dian Nirmala Sirait, Aditya Rifqi Fauzi, Ninditya Nugroho, Fadil Fahri, William Widitjiarso, Kristy Iskandar, Nurnaningsih

**Affiliations:** 1 Pediatric Surgery Division, Department of Surgery, Faculty of Medicine, Public Health and Nursing, Universitas Gadjah Mada/Dr. Sardjito Hospital, Jl. Kesehatan No. 1, Yogyakarta, 55281 Indonesia; 2 Department of Child Health, Faculty of Medicine, Public Health and Nursing, Universitas Gadjah Mada/UGM Academic Hospital, Yogyakarta, 55291 Indonesia; 3 Department of Child Health, Faculty of Medicine, Public Health and Nursing, Universitas Gadjah Mada/Dr. Sardjito Hospital, Yogyakarta, 55281 Indonesia

**Keywords:** Abdominal CT scan, Co-morbidities, Continuous clinical deterioration, Early recognition, Full-term neonate, Immediate surgical intervention, Late-onset of necrotizing enterocolitis, Risk factors

## Abstract

**Background:**

Necrotizing enterocolitis (NEC) is a common devastating inflammatory gastrointestinal disease and frequently occurs in premature infants. Here, we reported a case of late-onset NEC in a term neonate with good outcome after surgery for long-term follow-up.

**Case presentation:**

Ten-week-old male came to emergency unit due to prolonged diarrhea and abdominal distention. He was born at gestational age of 40 weeks with birth weight and Apgar score of 2800 g and 7/8, respectively. He had no history of formula feeding. Two weeks before admitted to the hospital, the patient had frequent diarrhea with fever. He was found lethargic with abdominal distention, absence of bowel sounds and abdominal tenderness. Plain abdominal x-ray and CT scan showed gastric and intestinal dilatation and gasless colon, suggesting a small bowel obstruction, and bowel wall thickening indicating peritonitis, without any free subdiaphragmatic air (pneumoperitoneum). Moreover, the patient did not have a congenital heart disease. While in intensive medical treatment, he showed a continuous clinical deterioration. All findings were suggestive of intestinal inflammation with clinical deterioration, and we decided to perform an emergency exploratory laparotomy and found an ischemia along the jejunoileal with a perforation at 25 cm above the ileocecal valve. Subsequently, we performed a double-barrel ileostomy through a separate incision from the laparotomy. Histopathological findings confirmed the diagnosis of NEC. We closed the stoma at postoperative day 43. The patient was discharged uneventfully a month after stoma closure.

**Conclusion:**

Abdominal CT scan might be useful to establish an early recognition of late-onset NEC; thus, immediate surgical intervention might be performed to decrease its morbidity and mortality. Moreover, late-onset NEC in term neonates might occur without any risk factors or significant co-morbidities.

## Background

Necrotizing enterocolitis (NEC) is one of the most common life-threatening conditions that affect the gastrointestinal tract. NEC occurrence is multifactorial and related to the immature gut barrier, disruption of bacterial colonization, intestinal ischemia, and inflammatory response [1–3]. Its incidence is approximately 1–3 in 1000 live births [4]. NEC usually occurs in premature infants with low birth weight. It frequently presents between 2 and 3 weeks of life in preterm infants, while NEC may appear within the first week of life in term infants [5]. NEC is classified into three staging according to the modified Bell criteria, which consists of systemic, intestinal, radiographic and laboratory findings [1].

Late-onset NEC in term infants is defined as the development of NEC after 7 days of life [6]. Interestingly, late-onset NEC shows a higher risk for mortality [6]. Many factors, including congenital heart disease, have been associated with NEC onset in term infants [6, 7]. While CT-scan is able to detect the pneumatosis intestinalis and perforation site [8], Epelman et al. suggest that CT scan might not be useful in clinical practice for evaluation of NEC [9]. Here, we reported a case of late-onset NEC in a term infant with good outcome after surgery for the long-term 3 years’ follow-up.

## Case presentation

**Fig. 1:**
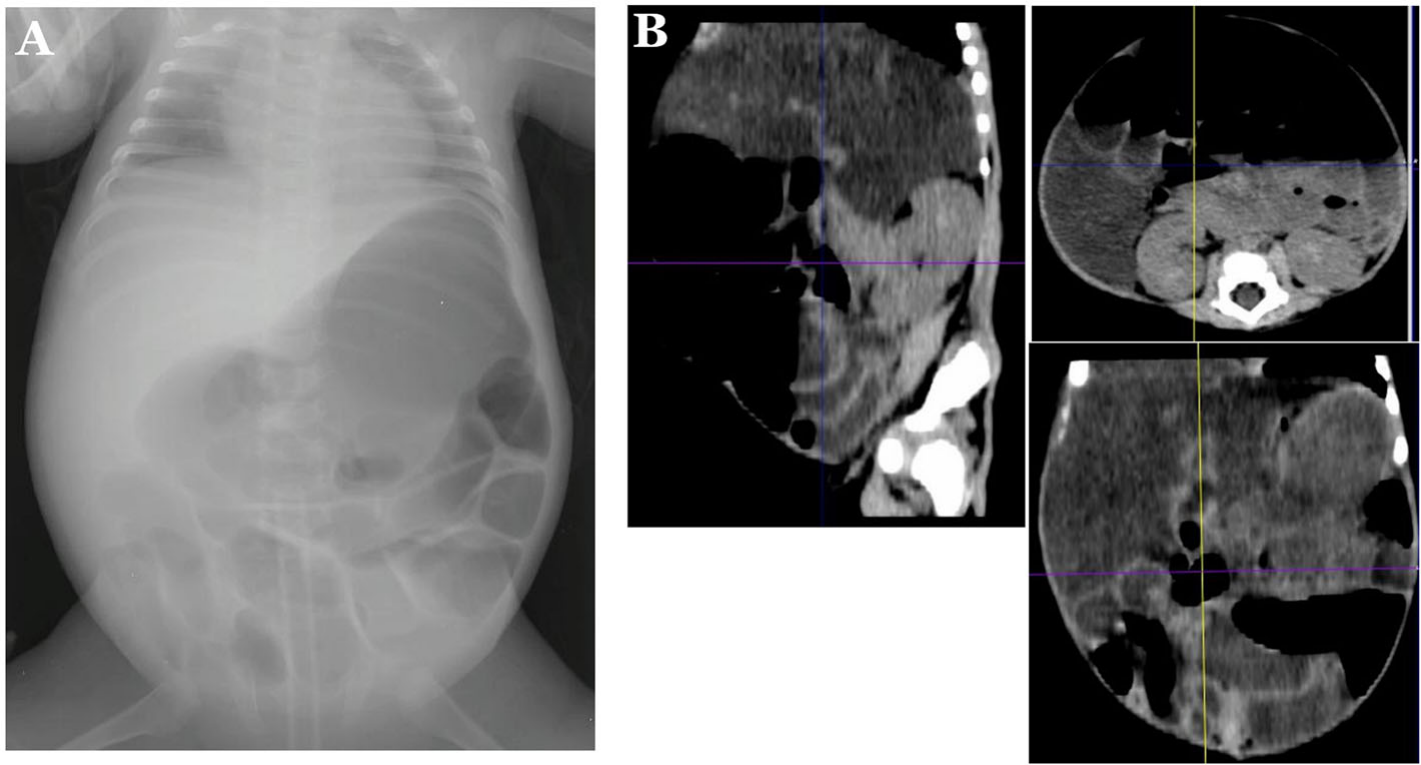
Plain abdominal x-ray (**a**) and CT scan (**b**) showed gastric and intestinal dilatation and gasless condition at the colon, suggesting a small bowel obstruction, and bowel wall thickening indicating peritonitis, without any free subdiaphragmatic air (pneumoperitoneum)

A ten-week-old male was brought to the emergency unit due to prolonged diarrhea and abdominal distention. He was born at gestational age of 40 weeks with birth weight and Apgar score of 2800 g and 7/8, respectively. He had no history of formula feeding. Two weeks before being admitted to the hospital, the patient had frequent diarrhea with fever. In the emergency unit, he was found lethargic and dehydrated. His vital signs were heart rate 143 beats per minute, respiratory rate 45 breaths per minute, temperature 37 °C, and lack of urine output. After 4 h rehydration program, his urine output was 1.6 mL/kg/hour. Laboratory findings were hemoglobin of 7.3 g/dL, white blood cell count of 7300/μL (differential white blood cell counts were as follows: neutrophil 58.5%, lymphocyte 23.2%, monocyte 17.9%), platelet of 73,000/μL, procalcitonin of 88.83 ng/mL, prothrombin time of 64.3/13.1 s, and activated partial thromboplastin time of 43/29.3 s. After he was resuscitated, he was then transferred into the ward. During 1 week in ward, while in intensive medical management, he showed a continuous clinical deterioration, including prolonged diarrhea accompanied by vomiting and abdominal distension. Laboratory findings were hemoglobin of 10.3 g/dL, white blood cell count of 16,490/μL (differential white blood cell counts were as follows: neutrophil 40%, lymphocyte 19%, monocyte 22%), platelet of 76,000/μL, procalcitonin of 1.56 ng/mL, prothrombin time of 37.3/12.5 s and activated partial thromboplastin time of 111.2/28.5 s. The patient, then, was consulted to us. He presented with abdominal distension, absence of bowel sounds and abdominal tenderness. His vital signs were heart rate 130 beats per minute, respiratory rate 34 breaths per minute, and temperature 37.2 °C, and lack of urine output for 6 h. After 4 h rehydration program, his urine output was 1.0 mL/kg/hour. He did not receive any pressor medicines such as dopamine, dobutamine or norepinephrine. Plain abdominal x-ray (Fig. 1a) and CT scan (Fig. 1b) showed gastric and intestinal dilatation with gasless condition at the colon, suggesting a small bowel obstruction, and bowel wall thickening indicating peritonitis, without any free subdiaphragmatic air (pneumoperitoneum). All findings were suggestive of intestinal inflammation with clinical deterioration. The patient did not have a congenital heart disease according to echocardiography. Moreover, there was not any maternal health issues during the antenatal maternal care. The mother had a routine antenatal care without any issues like diabetes, drug abuse, preeclampsia, or premature rupture of the membranes. The labour process took time about 29 h to complete the delivery.

**Fig. 2:**
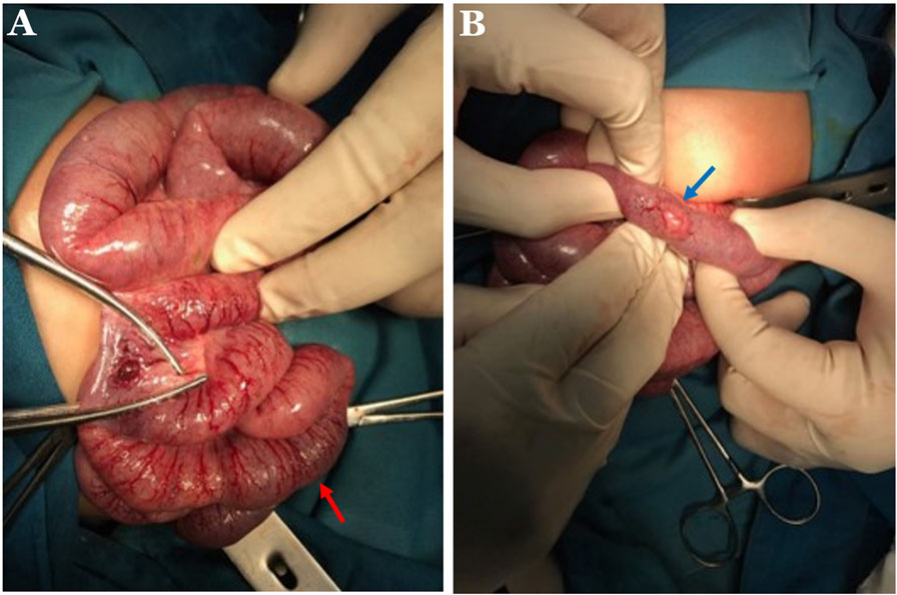
Intraoperative findings found an ischemia along the jejunoileal (**a**) and a perforation at 25 cm above the ileocecal valve (**b**)

**Fig. 3:**
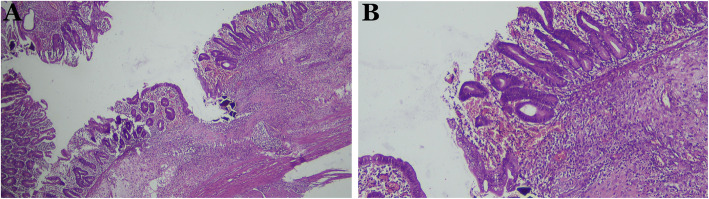
Histopathological findings revealed mucosal edema, hemorrhage and transmural necrosis, confirming the diagnosis of NEC. **a** original magnification, × 40. **b** × 100

We decided to perform an emergency exploratory laparotomy and found an ischemia along the jejunoileal (Fig. 2a) and a perforation at 25 cm above the ileocecal valve (Fig. 2b). Subsequently, we performed a double-barrel ileostomy through a separate incision from the laparotomy. After surgery, he was transferred into the pediatric intensive care unit (PICU). Laboratory findings were hemoglobin of 9.2 g/dL, WBC of 16,420/μL, platelet of 73,000/μL, and procalcitonin of 0.59 ng/mL. The stool culture result showed *Enterobacter cloacae*, while the blood culture result revealed no sign of any growth of bacteria. During the treatment at the PICU, he received cefotaxime, meropenem, metronidazole, fluconazole, transfusion of packed red blood cells 45 cc, fresh frozen plasma 45 cc, thrombocyte 50 cc, and albumin 25% 15 cc. We closed the stoma at postoperative day 43 due to high-output stoma and continuously imbalanced electrolytes, including serum potassium level of 3.0 mmol/L, sodium level of 129 mmol/L and chloride level of 95 mmol/L. The patient was discharged uneventfully a month after stoma closure. Moreover, histopathological findings confirmed the diagnosis of NEC, including mucosal edema, hemorrhage and transmural necrosis (Fig. 3).

There were no complications nor abdominal complaints until the last follow-up (approximately 3 years following stoma closure) when calling the parents during the preparation of this report.

## Discussion

Here, we reported a case of a term infant with late-onset NEC (i.e. 10-weeks-old) who is still showing a good outcome 3 years after surgery. In contrast, previous study revealed that late-onset NEC is a significant predictor for mortality in term infants [6]. Furthermore, they also found that most term infants with late-NEC have a congenital heart disease [6], but, this is not the case for our patient.

Our decision to perform emergency exploratory laparotomy was based on the following findings in our patient: 1) continuous clinical deterioration even with intensive medical treatment, and 2) general peritonitis indicated by physical examination (i.e. abdominal tenderness) and imaging, although without any pneumoperitoneum. Previous study reported that pneumoperitoneum only appears in < 50% cases of NEC with perforation [2].

Abdominal CT scan was able to detect peritonitis in our case, indicated by bowel wall thickening, although without any free subdiaphragmatic air (pneumoperitoneum). Therefore, we could intervene earlier by performing an exploratory laparotomy for our patient. There are several advantages of CT scan over other imaging methods, such as fast scan time, cross-sectional imaging for evaluation of intestinal wall and adjacent soft tissues, more affordable expense than magnetic resonance imaging, and without any sedation for pediatric patients [10, 11]. To the best of our knowledge, our case is the first report of evaluation of complication of late-onset NEC (i.e. peritonitis) using abdominal CT scan. However, notably, although CT scan was able to detect peritonitis in our case, Epelman et al. suggest that CT scan might not be beneficial in clinical practice for evaluation of NEC [9].

We performed a double barrel ileostomy on the perforation site without any resection. The choice of resection anastomosis or stoma for NEC patients depends on the local intestinal pathology and the patient’s general conditions [12]. We chose the stoma formation because of the ischemia all along the small intestines and worse general condition of our patient. We preferred to create the stoma through a separate incision from the laparotomy. No significant difference has been noted in stoma complications among different stoma locations [13]. Moreover, none of stoma types are considered superior to avoid stoma complications [14].

Early stoma closure (< 8 weeks) [15] was chosen because he had a high-output stoma and continuously imbalanced electrolytes. Following stoma closure, he showed better clinical outcome and eventually discharged from the hospital at 1 month after stoma closure. One current systematic review concluded that there was no difference of outcomes between early and late stoma closure in infants with NEC [15].

One of the differential diagnoses for our case was spontaneous intestinal perforation (SIP). SIP is a single intestinal perforation that is usually located at the terminal ileum and without any identifiable cause [16]. It is frequently seen in the pre-term infants with very low birth weight and extremely low birth weight, however, it can happen in the full-term infants with normal birth weight [16]. We diagnosed our case as NEC, not SIP, since besides a perforation at the terminal ileum, intraoperatively, we found an ischemia along the jejunoileal and histopathologically confirmed NEC. Another differential diagnosis for our case was ileal perforation due to intestinal ischemic process of unknown etiology without evidence of free air on imaging studies which may suggest perforation might have sealed off.

In conclusion, abdominal CT scan has an advantage to establish an early recognition of late-onset NEC, thus, immediate surgical intervention might be performed to decrease its morbidity and mortality. Moreover, late-onset NEC in term neonates might occur without any risk factors or significant co-morbidities.

## Data Availability

All data generated or analyzed during this study are included in the submission.

## Abbreviations

CT: Computed tomography
NEC: Necrotizing enterocolitis
